# Elevated prodromal psychotic symptoms lead to impaired social functioning via loneliness: A longitudinal mediation study

**DOI:** 10.1007/s00127-025-03004-0

**Published:** 2025-10-27

**Authors:** Louise Marcham, Thomas Richardson, Nicholas J. Kelley, Lyn Ellett

**Affiliations:** School of Psychology, University of Southampton, Southampton, SO17 1BJ UK

**Keywords:** Schizophrenia, Psychosis, Social functioning, Loneliness, Students, Prodromal

## Abstract

**Purpose:**

Although previous studies have considered loneliness as a mediator of the relationship between prodromal psychotic symptoms and impaired social functioning, there is lack of consensus regarding directionality of effects. We tested two competing hypotheses: Prodromal psychotic symptoms lead to deficits in social functioning via loneliness, vs. social functioning deficits lead to amplification of prodromal psychotic symptoms via loneliness.

**Methods:**

We implemented a longitudinal mediational design measuring variables (social functioning, loneliness and prodromal symptoms) at three time points over 6 to 8 months (*N* = 276) in a sample of British undergraduate students. We tested four longitudinal mediation path models across the three time points, controlling for age, gender and ethnicity.

**Results:**

Longitudinal mediational analyses suggest that both baseline prodromal symptoms and baseline distress about prodromal symptoms lead to small-to-moderate (standardized indirect effects = − 0.02) impairments in social functioning 6 to 8 months later via loneliness. However, baseline impairments in social functioning did not augment prodromal symptoms or symptom distress 6 to 8 months later.

**Conclusion:**

The results suggest that prodromal psychotic symptoms and distress about symptoms lead to impairments in social functioning via loneliness but not vice versa. These results suggest the need for preventative strategies to target loneliness which could prevent subsequent exacerbation of social functioning deficits. Future studies need to examine loneliness as a mechanism in the relationship between prodromal psychotic symptoms and social functioning across cultures, age groups, and over longer time periods.

## Introduction

Psychosis is amongst the leading causes of disability worldwide [1], with high treatment costs and relapse rates [2–4]. Early detection and intervention are therefore vital to implement preventative strategies and improve long term outcomes [5, 6]. A key focus in prevention is identifying prodromal psychotic symptoms in the general population, as these symptoms can predict transition to psychosis [7–10]. Up to 20% of the adult population can experience psychotic-like experiences without a diagnosis [11], and the first episode of psychosis is often preceded by a prodromal period [12–14]. The prodrome is defined as a period of experiencing gradual changes in thoughts, behaviours, perceptions, and functioning [15, 16]. These changes may include social withdrawal and reduced functioning, features which are themselves often considered part of the prodromal syndrome [17–19]. In the current study, we focus on the role of prodromal positive symptoms (and associated distress) as key features of the psychosis risk period and examine their longitudinal interplay with social functioning and loneliness. To clarify these conceptual overlaps, the present study explicitly examines both social functioning and prodromal positive symptoms as distinct, though interrelated, constructs. We test multiple longitudinal mediation models to explore their directional relationships, with loneliness as a potential pathway linking them.

Symptom-related distress is a key factor within the prodromal period [20], risk for psychosis is greater when individuals are distressed by their experiences, compared to those who are not [11, 21]. Symptom-related distress is a significant cause for seeking treatment and receiving a diagnosis of psychosis [22]. Distress often leads to social isolation which can reinforce paranoid beliefs and distress [23], exacerbating psychosis symptoms [14, 24]. Social impairment has been identified within the prodromal period and early stages of psychosis as a potential risk factor [16, 25–28], with social withdrawal reported as commonly occurring before psychosis onset [15, 29]. Individuals experiencing psychosis report reduced social networks and reduced social contact [30, 31]. Social isolation has been suggested to maintain psychosis symptoms due to lack of opportunities to disconfirm or review beliefs with others within social networks [32–34]. Social processes are therefore important to consider alongside prodromal positive symptoms, as potential targets for prevention strategies [14, 35].

Social functioning is defined as one’s ability to interact effectively across different social contexts such as work, education, leisure and family [36, 37]. Deficits in social functioning are a core feature of psychosis [38], leading to poor outcomes [37, 39]. Consequently, current interventions focus on improving social functioning for individuals with psychosis [40]. However, impairments in social functioning have been found long before psychosis onset [41, 42]. Poorer social functioning has been associated with greater prodrome symptom severity in groups at high risk of developing psychosis [43], and psychotic like experiences in the general population [44]. Early social functioning deficits are also predictive of psychosis onset [45–48], as well as worse social outcomes, such as increased isolation and loneliness [49, 50]. Longitudinal studies are therefore needed to explore the role of social functioning and loneliness in relation to prodromal positive symptoms and distress [41, 51].

Individuals with psychosis experience higher levels of loneliness compared to the general population [52], with positive symptoms associated with higher levels of loneliness [53]. It is suggested that loneliness impedes recovery by maintaining symptoms [28, 54]. Loneliness may also be a risk factor for psychosis onset [29, 55, 56]. Those at risk of developing psychosis report higher levels of loneliness compared to controls [43], and increased loneliness can lead to psychotic-like experiences within the general population [57–60]. Loneliness is a clinically relevant issue for the prodromal phase, due to its impact on physical and mental health outcomes for those who go on to develop psychosis [59, 61, 62]. In addition, loneliness may mediate between factors, such as childhood abuse and intimate partner violence, and psychosis symptom development and distress [55, 63, 64]. Loneliness has also been suggested to mediate between social functioning and health-related quality of life for those with psychosis [50]. Understanding the role of loneliness as a potential mediator, and in relation to prodromal experiences, is therefore important.

Increased loneliness has also been associated with poorer social functioning for those with psychosis [52], and poorer social functioning has been associated with increased experiences of loneliness for people with psychosis and for those experiencing prodromal positive symptoms [65–67]. Symptoms of psychosis may contribute towards loneliness [61, 62], with increases in paranoia found to increase experiences of loneliness within students [65] and psychotic-like experiences leading to increased experiences of loneliness [67]. However, these findings are not consistent, and social functioning deficits may precede symptom development [28, 68]. Despite the suggested relationships between these factors, there is a lack of research determining the nature and directionality of the relationships between social functioning, loneliness, and prodromal experiences within a non-clinical population [43, 67]. Understanding the relationships between social functioning, loneliness, prodromal positive symptoms and symptom distress may contribute towards the development of preventative strategies to reduce the risk of psychosis onset [13, 59, 60, 69].

Prior research suggests that loneliness mechanistically links prodromal positive symptoms/symptom distress to impaired social functioning [50, 52]. But there is a lack of consensus in the literature regarding directionality [43, 67]. On the one hand, elevated prodromal positive symptoms/symptom distress may lead to an increase in loneliness, which may in turn lead to impairments in social functioning via loneliness. On the other hand, baseline deficits in social functioning may increase loneliness which may then lead to the amplification of prodromal positive symptoms/symptom distress. Clarifying the directionality of these effects is important not only for theoretical understanding but also for informing clinical care. If social functioning deficits precede symptom expression, interventions may need to focus on improving social skills or expanding social networks. Conversely, if prodromal positive symptoms and associated distress initiate declines in social functioning, this would highlight the need for early identification of at-risk individuals and targeting loneliness as a modifiable pathway. We tested these two competing hypotheses using longitudinal mediation in a non-clinical sample where social functioning, loneliness and prodromal positive symptoms and distress were assessed at three time points: Time 1 (baseline), Time 2 (3–4 months), Time 3 (6–8 months).

## Method

### Participants and design

Participants were 276 first-year British undergraduate students aged 18 or older who participated in a wider study on tuition fee increases for British students [70]. Participants reported on their demographic characteristics (age, gender identity, ethnicity, disability and living situation) and then completed the measures described below at: Time 1 (baseline), Time 2 (3–4 months), Time 3 (6–8 months), see Table 1.

**Table 1:** Participant characteristics

Characteristic	Total/Time 1	Time 2	Time 3
*Age (years)*			
Mean (SD)	20.57 (5.37)	21.64 (5.42)	21.01 (5.36)
Range	18–58	18–59	19–59
*Gender*			
Female (Total %)	219 (79.3%)	177	174
Male (Total %)	56 (20.3%)	49	41
*Did not state*	1 (0.4%)	1	1
*Ethnicity*			
Asian/Asian British (Total %)	4 (1.4%)	4	4
Black/Black British (Total %)	3 (1.1%)	1	3
Mixed (Total %)	13 (4.7%)	7	11
Other (Total %)	2 (0.7%)	1	2
White British/White other (Total %)	251 (90.9%)	211	194
Did not state (Total %)	3 (1.1%)	3	2

### Procedure

Invitations to take part in the original study [70] were emailed to every university student union in the UK. Student unions were invited to forward the study details onto undergraduate students via emails, websites and/or social media. The study was advertised as a “Student Mental Health Survey,” looking at factors relating to mental health in students. Informed consent was gained prior to taking part, with optional entry to a lottery to win vouchers after taking part. Participants were invited via an email link to complete online surveys at each time point. Participants who did not complete multiple time points were excluded from the original study data. Of the 113 universities contacted, 46 advertised to the 2011 cohort and 44 advertised to the 2012 cohort. It was not known how many students saw the advert and therefore a response rate was not calculated.

The original study [70] recruited two cohorts of first-year undergraduate students and collected data across four time points. The current study analysed data starting from the second time point in the original study because the prodromal positive symptom measure was included at that point. Data was collected between June 2012 and January 2014 as follows: Time Point 1: August – September 2012 (cohort 1), February 2013 (cohort 2); Time Point 2: November – December 2012 (cohort 1), May – July 2013 (cohort 2); Time Point 3: February 2013 (cohort 1), November 2013 – January 2014 (cohort 2). At each time point participants completed: the Prodromal Questionnaire-Brief Version (PQ-B) [20], the three-item UCLA Loneliness Scale [71]; and the social functioning subscale of the RAND 36-Item Health Survey (RAND36-SF) [72].

### Prodromal positive symptoms and symptom distress

The PQ-B is a 21-item measure of prodromal positive symptoms of psychosis, such as perceptual abnormalities, unusual thought content, and suspiciousness (e.g., ‘do you feel that other people are watching you or talking about you?”) using a binary response format (Yes vs. No). The total prodromal positive symptom score ranges from 0 to 21 and is computed as the sum of all “yes” responses. For each endorsed item, participants also rate associated distress from 1 (*Strongly disagree*) to 5 (*Strongly agree*). The value of 0 is entered for distress items where there are no reported symptoms. A total distress score is calculated by the sum of all the distress ratings (range 0–105). This measure has good reliability for prodromal symptoms (*α* =.86) [73] and distress (*α* =.89) [73] and has been found to have concurrent validity with the Structured Interview for Prodromal Syndromes [6, 74]. Reliability for the study sample was good for prodromal symptoms (Time 1, *α* =.81) and distress (Time 1, *α* =.85). Although the PQ-B includes two additional items assessing social and academic/occupational functioning, these were explicitly excluded from the scoring algorithm [6] and were not used in our analyses.

### Loneliness

Loneliness was measured with a three-item version of the UCLA loneliness scale. Items included: “How often do you feel that you lack companionship?’, “How often do you feel left out?”, and “How often do you feel isolated from others”. Participants responded to the frequency with which they: (1) lack companionship, (2) feel left out, and (3) feel isolated from 1 (*Hardly ever*) to 3 (*Often*). Scores were summed such that total scores ranged from 3 to 9. This measure has acceptable reliability (*α* =.77) and correlates highly with the longer 20-item UCLA Loneliness Scale [75, 76]. Reliability for the study sample was good (Time 1, *α* =.86).

### Social functioning

Social functioning was measured with the social functioning subscale of the RAND 36-item health survey [72]. The social functioning subscale includes two items which ask “During the past 4 weeks, to what extent has your physical health or emotional problems interfered with your normal social activities with family, friends, neighbours, or and groups?” and “During the past 4 weeks, how much of the time has your physical health or emotional problems interfered with your social activities [like visiting with friends, relatives, etc.]?’). Participants responded to each of these items of a 5-point scale and responses were recoded to values of: 0, 25, 50, 75, 100. The average score for both questions is then calculated. Scores range from 0–100, where a lower score indicates the presence of limitations in social functioning. This subscale has been found to have good reliability (*α* =.85) [72]. The social functioning subscale also significantly correlates with other measures of social functioning [77]. Reliability for the study sample was good (Time 1, *α* =.86).

## Results

### Preliminary analyses

Of the 276 participants a majority (*N* = 163, 59.06%) had complete data and no participants missed more than one wave of data collection. Missing data was as follows: 4 (1.45%) participants did not complete Time 1, 49 (17.75%) participants did not complete Time 2, and 60 participants (21.74%) did not complete Time 3. Little’s missing completely at random (MCAR) test was not significant, *Χ*^2^ (24) = 24.42, *p* =.438, suggesting that our missing data was completely at random. Thus, we proceeded with Expectation-Maximization imputation of missing data. Demographic characteristics for the sample are shown in Table 1.The sample was predominantly female (79.3%, *n =* 219), and White British or White other (90.9%, *n =* 251). The age of participants ranged from 17 to 59 years (*M* = 21.01, *SD* = 5.36).

Prior to analysis, data were screened for missing values, outliers, or errors in inputting. Analyses were performed using SPSS (Version 29) [78]. Where any participants had completed at least 50% of the items for the measure, missing values were substituted with a mode [70]. Preliminary checks were conducted for suitability for bivariate correlations and mediation [79]. Q-Q plots indicated the assumptions of linearity and homoscedasticity were met. There were no collinearity problems observed. Normal distribution was assessed using Kolmogorov-Smirnov and Shapiro-Wilkes tests, alongside visual inspection of histograms. Variables with skewness or kurtosis outside of −2/+2 were considered to be outside of a normal distribution. The PQ-B distress measure had high kurtosis (4.17), although skewness was within the normal range (1.28). The PQ-B distress measure was kept as a continuous variable as it was required as a dependent variable and bootstrapping was applied to mediation analyses. The remaining variables were normally distributed. Boxplots were screened for outliers (i.e., three standard deviations from the means) and none were detected.

### Longitudinal mediation analyses

We examined competing models of the relationships among prodromal positive symptoms/symptom distress, loneliness, and social functioning via loneliness. One hypothesis is that elevated prodromal symptoms/symptom distress may lead to increases in loneliness, which may in turn lead to impairments in social functioning via loneliness. Another hypothesis is that baseline deficits in social functioning may increase loneliness which may then lead to the amplification of prodromal positive symptoms/symptom distress. We tested these two competing hypotheses using longitudinal mediation in a non-clinical sample where social functioning, loneliness and prodromal positive symptoms and distress were assessed at three time points: Time 1 (baseline), Time 2 (3–4 months), Time 3 (6–8 months). Correlations between social functioning, loneliness, positive symptoms, and symptom distress within and between time points are depicted in Table 2. Longitudinal mediation analysis were conducted in SPSS AMOS Version 30.

**Table 2:** Descriptive statistics and bivariate correlations between study variables across the three timepoints

Measure		M	SD	1	2	3	4	5	6	7	8	9	10	11
1	L (T1)	5.70	1.94	**―**										
2	L (T2)	5.70	1.79	0.70^***^	**―**									
3	L (T3)	5.61	1.81	0.58^***^	0.71^***^	**―**								
4	SF (T1)	76.89	25.38	− 0.52^***^	− 0.46^***^	− 0.37^***^	**―**							
5	SF (T2)	73.71	25.15	− 0.34^***^	− 0.53^***^	− 0.43^***^	0.48^***^	**―**						
6	SF (T3)	75.69	23.41	− 0.36^***^	− 0.42^***^	− 0.54^***^	0.54^***^	0.51^***^	**―**					
7	PS (T1)	3.77	3.51	0.40^***^	0.48^***^	0.38^***^	− 0.42^***^	− 0.32^***^	− 0.31^***^	**―**				
8	PS (T2)	3.25	3.25	0.36^***^	0.47^***^	0.40^***^	− 0.46^***^	− 0.43^***^	− 0.41^***^	0.78^***^	**―**			
9	PS (T3)	3.17	3.27	0.40^***^	0.53^***^	0.50^***^	− 0.41^***^	− 0.48^***^	− 0.43^***^	0.75^***^	0.82^***^	**―**		
10	D (T1)	11.51	12.90	0.43^***^	0.47^***^	0.40^***^	− 0.52^***^	− 0.34^***^	− 0.35^***^	0.94^***^	0.73^***^	0.68^***^	**―**	
11	D (T2)	10.15	11.45	0.41^***^	0.50^***^	0.42^***^	− 0.55^***^	− 0.46^***^	− 0.45^***^	0.75^***^	0.96^***^	0.78^***^	0.76^***^	**―**
12	D (T3)	9.90	11.73	0.43^***^	0.55^***^	0.54^***^	− 0.51^***^	− 0.55^***^	− 0.52^***^	0.72^***^	0.81^***^	0.95^***^	0.71^***^	0.83^***^

Note. L = Loneliness; SF = Social Functioning; PS = Prodromal Positive Symptoms; D = Symptom Distress. ***p <.001

### Model comparison and model selection

To examine the longitudinal mediation role of loneliness in the relationship between social functioning and prodromal symptoms we fit a series of path models. We used longitudinal mediation analysis to test directional hypotheses regarding these relationships over time. This approach is well suited for evaluating causal models involving temporal ordering, particularly when repeated measures are collected at appropriate intervals [80, 81]. First, we fit an autoregressive model in which each variable is regressed onto the proceeding observation of that variable (Model 1). Next, we added a longitudinal mediation effect from social functioning to positive symptoms via loneliness (Model 2). Then we tested the opposite longitudinal mediation effect from positive symptoms to social functioning via loneliness (Model 3). Finally, we tested a reciprocal model that included all paths from Model 1 and 2 (Model 4). All models controlled for age, sex, and ethnicity. All four models fit the data well for both positive symptoms (Table 3) and symptoms distress (Table 4). Of the four models we tested, the reciprocal model fit the data the best for both positive symptoms and symptom distress (See Table 5).

**Table 3:** The model fit information of different models for positive symptoms

Model	CFI	TLI	RMSEA	SRMR	X^2^	df	*p*
Model 1: Autoregressive Model	0.952	0.919	0.082	0.095	110.37	39	0.001
Model 2: Social Functioning to Positive Symptoms	0.966	0.933	0.075	0.071	85.7	34	0.001
Model 3: Positive Symptoms to Social Functioning	0.971	0.944	0.069	0.062	77.56	34	0.001
Model 4: Reciprocal Model	0.980	0.955	0.061	0.047	58.39	29	0.001

Note. CFI = comparative fit index; RMSEA = root mean square error of approximation; SRMR = standardized root mean square error; TLI = Tucker = Lewis index

**Table 4:** The model fit information of different models for symptom distress

Model	CFI	TLI	RMSEA	SRMR	X^2^	df	*p*
Model 1: Autoregressive Model	0.946	0.909	0.089	0.099	121.80	39	0.001
Model 2: Social Functioning to Positive Symptoms	0.961	0.925	0.080	0.068	93.34	34	0.001
Model 3: Positive Symptoms to Social Functioning	0.959	0.921	0.082	0.069	96.33	34	0.001
Model 4: Reciprocal Model	0.973	0.938	0.073	0.048	70.92	29	0.001

Note. CFI = comparative fit index; RMSEA = root mean square error of approximation; SRMR = standardized root mean square error; TLI = Tucker = Lewis index

### Longitudinal indirect effects

**Fig. 1:**
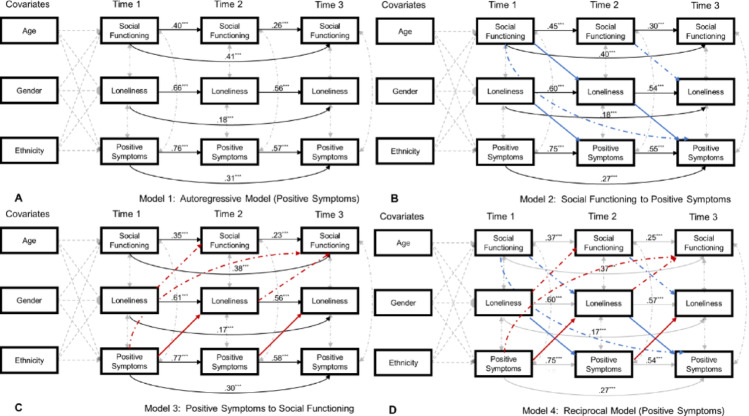
Longitudinal Mediation Models for Positive Symptoms. **Note.** Significant paths are in colour whereas non-significant paths are greyed out. Paths from Positive Symptoms to Social Functioning via Loneliness are noted in red. Paths from Social Functioning to Prodromal Positive Symptoms via Loneliness are noted in blue

***Social functioning*** $\to$ ***Loneliness*** $\to$ ***Positive symptoms.*** Social functioning at time 1 did not predict loneliness at time 2 (*β* = − 0.06, *p* =.236) but loneliness at time 2 did predict positive symptoms at time 3 (*β* = 0.15, *p* <.001). The standardized indirect effect of time 1 social functioning on time 3 positive symptoms via time 2 loneliness was not significant (*β* = − 0.001, *p* =.207, 95%CI [−0.004,0.001])^1^. See Fig. 1.

***Positive symptoms*** $\to$ ***Loneliness*** $\to$ ***Social functioning.*** Positive symptoms at time 1 predicted loneliness at time 2 (*β* = 0.19, *p* <.001) and higher loneliness at time 2 predicted worse social functioning at time 3 (*β* = − 0.11, *p* =.047). The standardized indirect effect of time 1 positive symptoms on time 3 social functioning via time 2 loneliness was significant (*β* = − 0.02, *p* =.030, 95%CI [−0.050, − 0.002]). See Fig. 1.

**Fig. 2:**
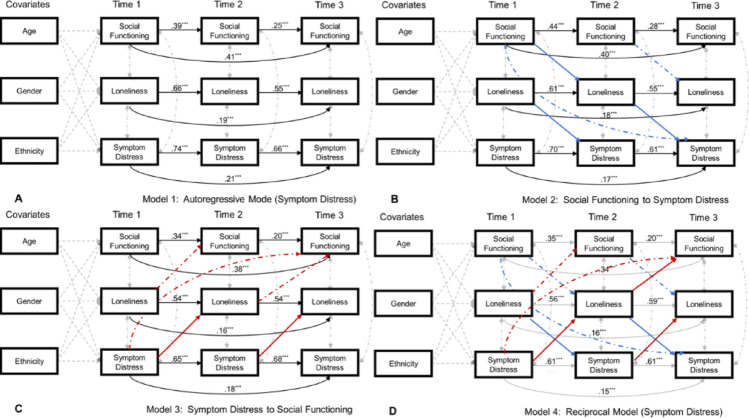
Longitudinal Mediation Models for Symptom Distress. **Note.** Significant paths are in colour whereas non-significant paths are greyed out. Paths from Symptom Distress to Social Functioning via Loneliness are noted in red. Paths from Social Functioning to Symptom Distress via Loneliness are noted in blue

***Social functioning *** $\to$ ***Loneliness*** $\to$ ***Symptom distress.*** Social functioning at time 1 did not predict loneliness at time 2 (*β* = − 0.03, *p* =.548) but loneliness at time 2 did predict positive prodromal symptoms at time 3 (*β* = 0.16, *p* <.001). The standardized indirect effect of time 1 social functioning on time 3 symptom distress via time 2 loneliness was not significant (*β* = − 0.01, *p* =.499, 95%CI [−0.03,0.01]). See Fig. 2.

***Symptom distress*** $\to$ ***Loneliness*** $\to$ ***Social functioning.*** Symptom distress at time 1 predicted loneliness at time 2 (*β* = 0.16, *p* =.005) and higher loneliness at time 2 predicted worse social functioning at time 3 (*β* = − 0.13, *p* =.025). The standardized indirect effect of time 1 symptom distress on time 3 social functioning via time 2 loneliness was significant (*β* = − 0.02, *p* =.016, 95%CI [−0.054, − 0.003]). See Fig. 2.

**Table 5:** Chi-squared difference tests and CFI differences for nested models

	Positive Prodromal Symptoms				Symptom Distress			
Model	ΔCFI	ΔX^2^	Δ*df*	*p*	ΔCFI	ΔX^2^	Δ*df*	*p*
Model 2 vs. Model 1	0.014	24.67	5	0.001	0.015	28.46	5	0.001
Model 3 vs. Model 1	0.019	32.81	5	0.001	0.013	25.47	5	0.001
Model 4 vs. Model 1	0.028	51.98	10	0.001	0.027	50.88	10	0.001
Model 4 vs. Model 2	0.014	27.31	5	0.001	0.012	22.42	5	0.001
Model 4 vs. Model 3	0.009	19.17	5	0.002	0.014	25.41	5	0.001

Note. CFI = comparative fit index; RMSEA = root mean square error of approximation; SRMR = standardized root mean square error

## Discussion

In the current study we tested competing hypotheses about the directionality of the associations among prodromal positive symptoms/symptom distress, loneliness, and social functioning in a non-clinical sample. The current research represents the first longitudinal study to simultaneously test whether impairments in social functioning exacerbate prodromal positive symptoms/symptom distress via loneliness or if elevated prodromal positive symptoms/symptom distress led to impairments in social functioning via increases in loneliness.

Social functioning was negatively associated with prodromal positive symptoms and symptom-related distress on the PQ-B, suggesting that as social functioning decreases, prodromal positive symptoms, and distress increases. Likewise, loneliness was positively associated with prodromal positive symptoms and symptom-related distress, suggesting that those reporting higher loneliness experience higher prodromal positive symptoms and distress. Collectively, these findings support existing research, that social functioning is lower for individuals experiencing prodromal positive symptoms [43], and loneliness is associated with prodromal positive symptoms [29, 43, 55, 56]. We add to the evidence by showing, for the first time, that lower social functioning, and higher levels of loneliness, are associated with higher prodromal distress. In line with previous studies, these findings suggest that social functioning impairments may present prior to diagnosis, preceding or alongside prodromal symptoms [41, 42].

We also tested a series of mediation models to examine whether loneliness mediated the effect of social functioning on prodromal positive symptoms and distress. Social functioning did not predict prodromal positive symptoms over time via loneliness. However, the reversed model was significant, suggesting that higher prodromal positive symptoms and symptom distress leads to lower social functioning via loneliness. While these results differ from research suggesting that social functioning deficits predict the onset of prodromal positive symptoms [65, 67] they are consistent with evidence that prodromal positive symptoms lead to lower social functioning over time [28, 68]. The results are also consistent with other studies highlighting the mechanistic role loneliness plays in the social functioning-prodromal positive symptom relationship [55, 63]. Additionally, our results are consistent with theorizing that prodromal positive symptoms can increase social withdrawal and reduce opportunities for social contact [23, 32, 33]. Evidence suggests that social changes take place before experiencing psychotic-like experiences [17–19], and that loneliness mediates between risk factors and psychosis development [55, 63, 64]. However, our findings suggest that prodromal positive symptoms and related distress lead to impairments in social functioning in a uni-directional model, via loneliness. Theories on psychosis symptom development may explain the significance of the reversed models as part of a maintaining cycle, as prodromal positive symptoms and distress can lead to increased loneliness and less opportunities to develop social skills, limiting opportunities for connections and support [23, 28, 32, 33]. This is consistent with cognitive models of psychosis which propose that early positive symptoms, such as suspiciousness or anomalous perceptions, may lead to distress and threat-based interpretations of social cues, increasing avoidance behaviors and social withdrawal. These behaviors can serve as self-protective mechanisms but ultimately reduce opportunities for meaningful social interaction, reinforcing feelings of loneliness and contributing to functional decline [23, 32, 33].

These findings suggest that prodromal positive symptoms and related distress might be important intervention targets for preventative strategies for psychosis. Targeting prodrome symptoms may enable a person to manage their experiences of these symptoms more effectively, reducing symptom related distress, thereby reducing loneliness and improving social functioning [32–34, 48]. The findings provide additional evidence that reduced social functioning and increased loneliness present alongside prodrome symptoms [48, 51, 69] and within non-clinical populations. Importantly, our findings highlight loneliness as a potentially modifiable pathway linking early symptoms to downstream social impairment, suggesting that interventions which reduce loneliness may help interrupt this progression. Public health strategies should therefore address these social determinants earlier in the course of symptom development [35]. Evidence suggests that community based social interventions can reduce loneliness [82] and, within the UK, social prescribing has been promoted and adapted to include online formats for accessibility [83]. Recent theoretical work suggests that the effectiveness of social prescribing may depend on whether it fosters social group identification, with interventions being most beneficial when they help individuals join and meaningfully identify with community groups that provide belonging and purpose [84]. Future research is needed to adapt and evaluate these identity-based approaches specifically for individuals experiencing prodromal positive symptoms, who may face unique barriers to group engagement and identification. Peer support groups can be an effective intervention for loneliness [85], and recent evidence suggests that digitally enabled peer support interventions may be especially effective and scalable. For example, participation in an online peer support program led to significant reductions in loneliness, depression, and anxiety over 90 days among a socially diverse adult sample [86]. Similarly, anonymous, synchronous, peer-moderated digital chats reduced momentary loneliness and increased optimism [87]. Peer-to-peer interaction plays a key role in digital interventions for psychosis, supporting engagement, perceived social support, and acceptability, particularly when interactions are moderated and interventions are co-designed with service users [88]. By fostering shared understanding and reducing stigma [89], these interactions may be especially well suited to address loneliness and prevent social withdrawal among individuals with emerging psychotic symptoms. Cognitive behavioural therapy has been delivered online to reduce loneliness and social anxiety, though it does not appear to improve general anxiety and depression symptoms [90, 91], thus the impact on prodromal psychotic symptoms is not known. Future work should explore how to adapt and evaluate these approaches within prodromal populations.

There are several limitations of the study that warrant consideration. Although a longitudinal design was used collecting data at three time points over an 8-month study period, this study was not able to determine whether these relationships were maintained longer-term. The PQ-B distress measure was also outside of normal distribution, and therefore did not meet some of the assumptions for analysis and could have limited the results. The study may also be limited by the small sample size and therefore larger scale studies may be required to determine if the findings are replicated. The sample for this study also mostly comprised of White British, female, young adults, from a high-income country, which could limit generalisability to males and may not generalise cross-culturally and for low or middle income countries. The study also recruited from a student population which is not representative of the general population. Furthermore, as the original study was advertised as a mental health survey, it may have attracted participants who were more likely to have poor mental health. Future research might usefully determine whether these findings are replicated within other populations, including cross-culturally, and across a longer period of time. Future studies should also examine whether these findings are replicated for individuals at ultra-high risk of psychosis. In addition, as the average age of psychosis onset may be 20.5 years [92], future studies focusing on prevention might also explore whether these findings hold in younger adolescent populations.

## Conclusion

This is the first study to show that loneliness acts as a mediator between prodromal positive symptoms and symptom-related distress, and social functioning within a non-clinical population. Public health interventions should target prodrome symptoms and related distress, with an aim to reduce loneliness and increase opportunities to develop social functioning skills. Future longitudinal studies are needed to determine whether these findings hold within ultra-high risk and younger populations, and whether the findings generalise cross-culturally.

## Data Availability

The dataset used is available from TR on reasonable request.

## Footnotes

1 We estimated confidence intervals using the bias-corrected percentile method and 5,000 bootstrap samples.
